# How Self-Regulation and Executive Functions Deficits Affect Quality of Life of Children/Adolescents with Emotional Regulation Disorders

**DOI:** 10.3390/children10101622

**Published:** 2023-09-28

**Authors:** Ginan Hammud, Ayelet Avital-Magen, Guy Schusheim, Inbar Barzuza, Batya Engel-Yeger

**Affiliations:** 1 Department of Occupational Therapy, Faculty of Social Welfare and Health Sciences, University of Haifa, Haifa 3498838, Israel; ghammud@campus.haifa.ac.il; 2 Child and Adolescent Mental Health Clinic, Haemeq Medical Center, Afula 1834111, Israel

**Keywords:** self-regulation, executive functions, quality of life, children/adolescents

## Abstract

Background: Deficits in self-regulation and executive functions (EFs) frequently characterize children/adolescents with emotional regulation disorders and restrict their daily function and quality of life (QOL). These deficits are mainly manifested by neuropsychological measures in laboratory settings. This study aimed to compare self-regulation and EFs by ecological measures to reflect the implications in daily life between children with emotional regulation disorders and healthy controls and examine the relations between self-regulation, EFs and QOL in the study group. Methods: the participants were 49 children aged 8–18: 25 children/adolescents with emotional regulation disorders and 24 healthy children. The parents completed a socio-demographic questionnaire, the Child Behavior Checklist (CBCL), the Behavior Rating Inventory of Executive Functions (BRIEF) and the Pediatric Quality of Life Inventory (Peds-QL). Results: The study group had greater self-regulation difficulties (internalization and externalization problems), executive dysfunctions (EFdys) (including metacognition difficulties) and a lower QOL. Their internalization and externalization problems correlated with reduced EFs and QOL. Internalization predicted the physical and emotional QOLs, while metacognition predicted social and school-related QOLs. Conclusions: Deficits in self-regulation and EFs are prevalent in children/adolescents with emotional disorders and restrict their daily function and QOL. Therefore, they should be routinely evaluated by ecological instruments to reflect daily restrictions.

## 1. Introduction

Emotional regulation disorders are prevalent among 2.6–6.5% of children and adolescents worldwide [1] and may linger into adulthood [2,3].

According to the criteria of *The Diagnostic and Statistical Manual of Mental Disorders, 5th edition* (DSM-V) [4] and The International Classification of Diseases (ICD) [5], emotional regulation disorders in children and adolescents can be classified as internalization problems (such as anxiety and depression), or externalization problems (such as rule breaking or aggressive behavior) [6] that appear late in childhood and negatively impact various aspects of the daily life of the individual and his/her family [7]. Emotional regulation disorders can lead to higher rates of school absence, impaired academic performance, reduced social interactions and even higher manifestations of suicidal behavior [8,9,10]. Emotional regulation problems can occur in adolescence age as a result of experiencing violence, such as school bullying that is related to sibling victimization and sibling bullying [11]. Studies highlight that experiencing sibling bullying in childhood may lead to anxiety and depression [12]. Moreover, other risk factors related to EFdys include exposure to illness at a younger age, physical stress, alcohol, drugs, nutrition, infections, a lifetime history of environmental exposures to toxins, stress, as well as the social environment and stressful life events, maltreatment and family member mental illness (maternal depression in particular) [13,14]. Therefore, emotional regulation disorders should be diagnosed as early as possible with a deep understanding of their characteristics and their functional and developmental implications.

One core factor that is significantly impaired in emotional regulation disorder is *self-regulation*. Self-regulation represents the ability to manage emotions, cognitive functions and behavior and encompasses the coping skills we use to effectively pursue and refine goals to manage life events [15,16]. Self-regulation difficulties include internalization problems, which are conditions characterized by disordered moods or emotions (such as being withdrawn, anxiety, depression or somatic complaints) and externalization problems, which are conditions characterized by dysregulated behavior (such as rule breaking or aggressive behavior) [6]. Self-regulation difficulties are prevalent in children with emotional regulation disorders and have a significant impact on behavior and daily function [16]. One of the measures used to reflect these outcomes was the Child Behavior Checklist (CBCL) [6,17,18,19].

Another significant aspect of emotional regulation is the ability to monitor and modulate emotions [20] and create adaptive behavior [16,21]. These abilities are dominated by the high cognitive processes named *Executive Functions* (EFs). EFs include attention, planning, working memory, impulse control, inhibition, shifting, organization, problem solving, initiation and action monitoring [22,23]. EFs are fundamental for performing almost all daily life activities, for proper social interactions and for academic achievements [24,25,26,27]. Although EFs mature during adolescence, they start to develop in early childhood, parallel to the development of the frontal lobe. Significant improvement in EFs occurs during the school years [28].

EF impairments are prevalent in children and adolescents with emotional regulation difficulties. Studies report on impairments in working memory, planning, shifting, in the ability to maintain attention and inhibit distractions or irrelevant information [29,30,31,32,33,34]. These EF difficulties are associated with a child’s reduced academic performance, elevated anxiety and emotional overload, poor sleep quality and substance use, including alcohol and cannabis use [35,36,37]. These outcomes were found to continue with age into adulthood. Difficulties in EFs further enhance social problems [27,38], deteriorate academic performance, daily function and independence in children with emotional regulation disorders, and contribute to their limited QOL [39,40,41,42,43,44].

*Quality of life* (QOL) is defined as a person’s subjective feelings about environmental conditions, illness, level of functioning and enjoyment of life activities [45]. The World Health Organization Quality of Life Group [46] adds that QOL is the person’s perception of his or her status in life in the context of the culture and value systems in which he or she lives and in relation to his or her goals, expectations, standards and concerns. QOL refers to several domains: physical, social, independence, environment, spirituality, religion and personal beliefs). Based on the WHO, QOL is one of the main outcome measures of intervention efficacy [47].

Nonetheless, with regard to emotional regulation disorders, interventions frequently focus on improving the symptoms and not on the QOL and the functional consequences of the disease [48]. Therefore, studies should further illuminate the implications of emotional regulation disorders in children in their daily lives and consider the impacts of the known comorbidities as EFdys on QOL. This is in line with the International Classification of Functioning (ICF) framework of the World Health Organization [47], which stresses the need to refer in intervention to the interactions between the health condition, the ability to perform daily activities and participate in daily life settings. By referring to daily life and not only to what is happening in the clinic, intervention may better fit the individual’s function in a real-life context, expectations and needs and, by that, elevate their QOL [47]. Yet, with regard to children/adolescents with emotional regulation disorders, an evaluation is mainly based on clinical measures. Ecological valid measures that reflect how the health condition affects function in real life are hardly in use [49]. Based on the far-reaching negative effects of emotional regulation disorders and prevalent EFdys on a child’s development, function and QOL [7,50,51,52], and in line with the ICF, the present study aimed to elaborate the knowledge about self-regulation difficulties and the expressions of EFdys on a child’s daily life and examine their relation to QOL. The results of this study may shed light on expressions of self-regulation difficulties, EFdys in the daily life of children/adolescents with emotional regulation disorders, on their relations to various quality-of-life domains and on the feasibility of evaluation tools relevant to these purposes. This knowledge may improve evaluations and interventions for children and adolescents with emotional regulation disorders, with a sensitive focus on specific real-life domains, which should be improved in order to optimize health and well-being. For that, this study aimed to (1) compare the rates of self-regulation difficulties and EFdys, as expressed in daily life scenarios and QOL, with respect to physical, emotional, social and school domains between the study group and the control group. (2) Examine the relations between the self-regulation difficulties, EFs and QOL in the study group. (3) Examine the relative contribution of self-regulation and EFs to the prediction of QOL among the study group.

Hypotheses: 1. Children/adolescents with emotional regulation disorders would show significantly higher rates of self-regulation difficulties and EFdys compared to the control group. The QOL of children/adolescents with emotional regulation disorders would be significantly lower than the healthy controls. 2. The greater the self-regulation difficulties, the greater EFdys and the lower QOL of the study group. 3. The severity of the difficulties in self-regulation and EFs would predict the QOL of the study group.

## 2. Materials and Methods

### 2.1. Participants

This study included 49 children and adolescents aged 8–18 years and their parents. The study group comprised 25 children and adolescents, 14 boys and 11 girls, diagnosed with emotional regulation disorders by psychiatrists, according to the DSM-5 and a clinical interview (see Table 1). The control group included 24 children and adolescents, 14 boys and 10 girls, with typical development and normal emotional health/self-regulation, according to their parents’ report and their normal scores on the Child Behavior Checklist (CBCL) [6]. Both groups were matched by age and gender but significantly differed in maternal education (χ^2^ (2) = 20.75, *p* < 0.001) and in socioeconomic status (χ^2^ (2) = 22.84, *p* < 0.001). Yet, both parameters were significantly positively correlated (r = 0.47, *p* = 0.001). Therefore, statistical adjustments were performed, as will be further described.

The exclusion criteria for both groups included serious health conditions/chronic illness (except for the psychiatric conditions), uncorrected sensory impairment and cognitive deficits (participants had a normal IQ according to the parents’ reports).

### 2.2. Measures

#### 2.2.1. Socio-Demographic Questionnaire

A socio-demographic questionnaire was used to gather information about the age, sex, socio-demographic status of the family, child/adolescent health status, medication or other treatments. The questionnaire was a screening tool used for including the child/adolescent and his or her parents in the study.

#### 2.2.2. Child Behavior Checklist (CBCL) [6]

A standard measure for children and adolescents ages 4–18 that parents fill in to describe their child’s self-regulation problems [19] is related to emotional and behavioral problems [17] and includes items that describe abilities and problems. This measure consists of two parts: a part that relates to the social function and a part that deals with emotional-behavioral problems (behavioral profile). In the present study, only the behavioral part—which includes 113 statements—was used. The parent marks answers on a scale from 0 to 2 (0 = the item is not true for my child, 1 = to some extent/sometimes true, and 2 = the item is very true or often true) based on the last 6 months. The CBCL scores are divided into internalizing, externalizing and total scores. The internalizing score includes emotional problem scales: anxious/depressed, withdrawn/depressed and somatic complaints. The externalizing score includes behavioral problem scales: the rule-breaking behavior and aggressive behavior syndrome scales. Three other syndrome scales were social problems, thought problems and attention problems. The clinical cross-sectional score is above 63 according to a total T-score. A score between 60 and 63 indicates borderline function; below 60 is considered normal. This measure has a high repeat test reliability of 0.9, a good internal consistency of 0.72–0.97 and criterion validity [6]. In the present study, the CBCL was used to verify and characterize self-regulation difficulties in the study group and rule them out in the control group.

#### 2.2.3. Behavior Rating Inventory of Executive Functions (BRIEF) [53]

The BRIEF is a behavioral rating measure for children and adolescents aged 5–18, designed to examine EFs as expressed in daily life scenarios (for example, overreacts to small problems, leaves his environment “messy”) according to the parents’ report on their child’s behavior in his/her daily natural environment. The BRIEF contains two parts: the BRI, behavioral regulation (composed of the inhibition, shifting and emotional control scales), and the metacognition, MI (composed of the initiation, working memory, planning, organization of materials and monitoring scales). A total score, the global executive composite (GEC), is also calculated. There are 86 statements describing diverse behaviors. Parents rate the frequency of these behaviors on a Likert scale from 1 to 3 (1 = infrequently, 3 = often). The overall score is converted to a standard score. A score of 65 or higher indicates deficiencies in EFs. The BRIEF has good psychometric properties [53,54].

#### 2.2.4. Pediatric Quality of Life Inventory (Peds-QL) [55]

The Peds-QL measure examines the QOL associated with health in patients or in healthy populations of children and adolescents. The Peds-QL has two versions: a version that relies on parental reporting and the other on a child’s self-reporting. In the current study, parental reporting was used. The Peds-QL includes four domains of QOL in the context of 1. physical functioning (eight items), 2. emotional functioning (five items), 3. social functioning (five items) and 4. school functioning (five items). The questionnaire filler rates the frequency with which his/her child had difficulty in the last month on a 5-point Likert scale in all items (0 = never difficulty, 1 = almost never difficulty, 2 = sometimes there is difficulty, 3 = often there is difficulty, 4 = there is almost always a difficulty). The scores are converted to a scale from 0 to 100 (0 = 100, 1 = 75, 2 = 50, 3 = 25, 4 = 0). A higher overall score indicates a higher QOL. The Peds-QL has good psychometric properties [55].

### 2.3. Procedure

The current study was conducted in accordance with the Declaration of Helsinki Committee (blinded) with the approval code 0174-17-EMC and approval date of 17 December 2018 and according to the Faculty of Social Welfare and Health Sciences Ethics Committee, the University of Haifa (approval code: 333/18; approval date: 17 September 2018).

After receiving ethics approval from the University of Haifa and from the Helsinki Committee, the study group was recruited via advertisements published in their ambulatory psychiatric clinic. The control group was recruited via similar advertisements published in neighborhoods in the same geographic area and from the immediate environment of the researchers. Parents and children who wished to participate in this study contacted the study conductor via phone, and a meeting was set with them at the clinic or in their homes. In this meeting, parents and their children signed the consent form, while the young children approved their consent orally. Parents to all children/adolescents completed the CBCL, BRIEF and the Peds-QL measures.

### 2.4. Data Analysis

The data were analyzed using SPSS-27. After analyzing the descriptive statistics and testing normality, chi-square analysis and t-tests examined the differences between the groups in terms of the socio-demographic parameters and the prevalence of EFdys. The differences between groups in the dependent variables were examined by multivariate analysis of covariance (MANCOVA) for the questionnaires’ scales and by analysis of covariance (ANCOVA) for the questionnaires’ total scores when controlling the socioeconomic status (because maternal education correlated with familial socioeconomic status). Effect sizes were calculated using partial eta square (η_p_^2^ = the sum of squares of an effect for one variable/(the sum of squares of an effect for one variable + the sum of squares error) in the ANCOVA and MANCOVA) [56,57]. The magnitude of the partial eta square is explained according to Cohen’s suggestion (1988): the partial eta square 0.01 is a small effect, 0.06 is a moderate effect, and 0.14 is a large effect [58]. Stepwise linear regression examined the relative contribution of the CBCL and EFs components to the prediction of QOL. The significance level was set at *p* ≤ 0.05.

### 2.5. Study Design

This is a cross-sectional and correlative study. Cross-sectional studies analyze data gathered from a population at a single point in time for measuring the prevalence of health outcomes and for describing features of a population [59]. This design enabled us to perform this observational study and to gather data to characterize self-regulation and EFs and QOL among children/adolescents with emotional regulation disorders at a single point in time and compare this information to that of the health controls. The correlative part enabled us to examine the relations between the examined variables among the study group in order to understand the implications of EFdys on various QOL domains.

## 3. Results

### 3.1. Hypothesis 1: Differences between Both Groups

When referring to the differences in self-regulation, as mentioned above, the control group included children with no self-regulation difficulties according to the CBCL cutoff score. Table 2 depicts the CBCL scores of both groups to reflect the greater self-regulation difficulties of the study group, as manifested in all CBCL scales, in the internalization, externalization and total scores. The effect size of all observed variables was the partial eta square η_p_^2^ that ranged from 0.14 to 0.45, indicating large effect sizes.

The differences in EFs between both groups according to the BRIEF:

The study group showed greater EFdys than the control group in all EFs components, as reported by parents in the BRIEF (except for the ability to organize materials), as presented in Table 3. The effect size of all observed variables was the partial eta square η_p_^2^ that ranged from 0.05 to 0.42, indicating small to large effect sizes.

Chi-square analyses showed that EFdys (based on a total standard score of 65 and above) were found among 60% of the study group, as compared to 0% of the controls (χ_(1)_^2^ = 20.75, *p* < 0.0001).

The differences in QOL domains between both groups according to the Peds-QL:

The study group had significantly lower emotional, social and school-related QOLs than the control group. The effect size of all the observed variables was the partial eta square η_p_^2^ that ranged from 0.05 to 0.46, indicating small to large effect sizes. (see Table 4).

### 3.2. Hypothesis 2: Correlations between Variables in the Study Group

Medium to high significant positive correlations were found between the BRIEF components and CBCL scales. Therefore, greater difficulties in self-regulation (CBCL) correlated with greater EFdys (BRIEF). Medium to high significant negative correlations were found between the CBCL scores and Peds-QL scores: greater difficulties in self-regulation correlated with a lower QOL (see Table 5).

### 3.3. Hypothesis 3: Prediction of QOL in the Study Group by Self-Regulation (CBCL) and EFs (BRIEF)

As presented in Table 6, internalization difficulties significantly predicted 39.9% of the physical QOL. That means the more severe the difficulties in internalization, the lower the physical QOL. Internalization difficulties also predicted 58% of the emotional QOL, while the BRI score (BRIEF) added 8% to the variance. The MI score (BRIEF) significantly predicted 25.8% of the social QOL and 51.9% of the school-related QOL. The MI score contributed 64.6% to the psychosocial QOL, while the internalization score added 12.2% to this prediction. Internalization contributed 65% to the prediction of the total QOL, while the BRI score added 7% to this prediction (see Table 6). Hence, among the study group, difficulties in internalization predicted lower physical and emotional QOLs. Difficulties in emotional regulation, as manifested by the BRIEF-BRI, contributed to the reduced emotional and total QOLs. Difficulties in metacognition, as measured by the BRIEF-MI, predicted lower social, psychosocial and school-related QOLs.

## 4. Discussion

The present study aimed to compare self-regulation, EFs, as expressed in daily life scenarios, and QOL between children/adolescents with emotional regulation disorders and healthy controls and examine the relations between the variables in the study group. The data were gathered from parents, as they are the main caregivers who provide authentic information about the child’s real life, using ecologically valid measures.

The first hypothesis was confirmed. As expected, based on the CBCL, greater self-regulation difficulties, including internalizing and externalizing symptoms, were found among children/adolescents with emotional regulation disorders compared to the control group. The CBCL was used in previous studies to profile self-regulation difficulties in clinical populations, such as children with disruptive behavior [60] and anxiety disorders [61]. Profiling self-regulation difficulties during childhood is critical because it enables early intervention and a reduction in the negative impacts that appear later in life, such as substance use [62], suicidality [63], impaired social functioning [64], and a higher risk of developing psychiatric disorders such as depression [65]. The present study supports the feasibility of the CBCL as a screening tool for emotional regulation disorders in children and adolescents.

The present study provided an elaborated point of view and explained the regulation difficulties by difficulties in EFs, as reflected by the BRIEF. Difficulties in EFs were significantly more prevalent among the study group in all EF components (except for organizing materials). Previous studies showed that children and adolescents with emotional regulation disorders, such as oppositional defiant disorder (ODD), conduct disorder (CD) and depression, had greater EF difficulties in inhibition [66,67]. The existing literature focuses mainly on specific EF components, also measured in the lab by neuropsychological assessments [68,69], while the present study, which used the BRIEF, referred to all EF components. Although children and adolescents with cognitive deficits were excluded from this study, EFdys were found to be prevalent in the study group, enhancing that high cognitive abilities such as EFs could be impaired, as reported by the parents on daily life functioning.

Knowledge about EFs and their implications on a child’s academic performance, daily function, development and QOL is crucial for the health care provided to children and adolescents with emotional regulation difficulties [70,71]. First, this is because EFs play a major role in the pathogenesis of this population, and second, a detailed evaluation of EFs in daily life may contribute to the explanation of behavioral problems and related functional restrictions. This understanding may assist in focusing interventions on improving EFs with regard to a real-life context. Recent studies suggest multiple efficient interventions for treating EFdys in children and adolescents, such as exercise interventions, especially chronic sessions of exercise interventions with moderate intensity [72], implemented in curricular or sports and physical activity program settings [73]; cognitive training [74]; virtual reality-based interventions [75]; and mindfulness meditation [76]. EF skills can also be treated through scaffolded training strategies by mitigating disruptive bottom-up influences such as stress, as well as by training EF skills and adding a reflective, metacognitive component to promote a further transfer of the learned skills [77]. By combining ecologically valid evaluations such as the BRIEF, involving parents in the evaluation process, and referring to the child’s and family’s real-life scenarios, intervention may be more efficient. The present study suggests that BRIEF is a feasible tool for screening EF difficulties in this population, and its functional information may assist in tailoring the intervention based on the child’s function in a real-life context.

When referring to QOL, while the physical QOL did not differ between the groups, gaps were found in the social, emotional and school-related domains. Indeed, children/adolescents with emotional regulation disorders face substantial challenges in a social life context; they experience peer rejection [78] and have poor relationships with their peers [79], which in turn is known to lead to loneliness [80]. In school-aged children and during adolescence, social interactions are critical for their emotional well-being, beliefs about the self and academic performance [81]. This may explain the increased risk of having future isolation [82], antisocial outcomes and criminal charges [83]. These challenges may worsen their psychological functioning and the prognosis of their mental health status; therefore, it is necessary to explore how the disorder and related self-regulation and EFs affect a child’s social life in order to provide intervention when needed and thus promise a child’s optimal development and well-being.

With regard to the second hypothesis, significant correlations were found between the BRIEF and CBCL scores. According to Woltering, Lishak, Hodgson, Granic and Zelazo (2016) [84], EFdys underlie externalizing problem behaviors in children with disruptive behavior, for example, reduced inhibitory control correlated with higher externalizing symptoms [85,86]. Furthermore, EFdys were found to predict the existence of externalizing problems over time [87]. Therefore, it is important to evaluate and treat EFdys appropriately as soon as possible and understand their relation to other comorbidities and their implications on a child’s daily life. Support for this may be found in the further significant correlations between self-regulation, EFdys and QOL. The existing literature presents similar outcomes. For example, associations were found between self-regulation difficulties, as measured by the CBCL, and the physical and school-related QOLs [88]. In other clinical populations, such as children with autistic spectrum disorders (ASD) or with obsessive-compulsive disorder (OCD), externalizing and internalizing symptoms reflected in the CBCL predicted a reduced QOL [89,90]. The present study strengthens the previous report that internalizing symptoms is associated with a reduced QOL [91].

In regard to the third hypothesis, when predicting specific QOL domains, the main predictor of physical and emotional QOLs was the CBCL score of internalization, while the BRIEF-MI score was the main predictor of social and school-related QOLs. Studies found relations between the internalization/externalization symptoms and a child’s lower QOL [92,93,94]. Yet, in the present study, externalization symptoms did not contribute significantly to the prediction of any of the QOL domains. The fact that anxiety, which is an internalizing disorder [95], was the prevalent diagnosis of our sample may explain this result. Nonetheless, studies indicate that externalizing symptoms may lead to low life satisfaction in adolescence [96] and affect academic achievements and daily functioning [97]. Therefore, further studies on larger sample sizes should examine the implications of externalizing symptoms on a person’s daily function and QOL in order to apply this knowledge in interventions and enhance a child’s development and well-being.

To summarize, the far-reaching effects of self-dysregulation and EFdys on development and their frequent persistence in adulthood [98,99,100,101] highlight how important it is to screen and treat these cardinal factors in children with emotional regulation disorders as early as possible [102]. Based on the family-centered approach [103], it is important to gather information from the parents, who are the child’s main caregivers. Also, it is recommended to apply valid ecological evaluations, such as the BRIEF, that reflect daily life in order to better understand the disease characteristics and comorbidities implications on a person’s function in real-life settings. By that, clinicians may elevate parents’ awareness of the difficulties that their children face in daily life. As a result, parents may be more sensitive to their children’s specific needs, may look for relevant health care, and apply coping strategies to improve their family relations and their child’s optimal function, development and well-being. The current study has theoretical and practical implications. Theoretically, it emphasizes the relevance of applying the ICF model to children/adolescents with emotional regulation disorders. Specifically, this study highlights the importance of referring to EFs as a major part of the pathogenesis of emotional regulation disorders and functional restrictions. Practically, this study highlights the relevance of evaluating EFs, using ecologically valid evaluations, and providing a detailed picture of EFs and their relations to daily life. Clinicians should encourage the involvement of parents in the evaluation and intervention processes because they can provide the most relevant information on their child’s functioning in daily life. Intervention should also elevate the awareness of researchers, individuals struggling with emotional regulation disorders, family members and health professionals to the involvement of self-regulation and EF difficulties in children with emotional regulation disorders and their relation to the child’s daily life, as expressed in their QOL. Moreover, a multidisciplinary intervention is recommended, in which professionals such as psychiatrists and occupational therapists work together to improve EFdys and self-regulation with application to the child’s function in natural environments. This may elevate the intervention outcomes, child and parents’ involvement, and by that, optimize the child’s health, development and well-being.

## 5. Conclusions

Self-regulation difficulties, EFdys and a reduced QOL are prevalent among children/adolescents with emotional regulation disorders. Worse self-regulation and EFs reduce a child’s QOL. The relationship between these factors and their negative implications on a child’s daily life should be further explored, especially in the critical developmental stages, such as childhood and adolescence. This study supports the relevance of applying the ICF-CY model to intervention programs for children/adolescents with emotional regulation disorders. Together with the applications of ecological measures, clinicians may improve intervention outcomes, i.e., reduce symptoms and distress and improve development and well-being. This approach may enhance the parents’ and children’s involvement in therapy and lead to better intervention results, such as shortening the duration of the treatment, reducing the costs of health services and, most importantly, increasing the child’s health, QOL and optimal development.

## 6. Research Limitations

The present study should be considered in light of the following limitations: the relatively small sample size of participants may restrict the generalization of the findings. Both groups differed in specific socio-demographic parameters. Thus, it is recommended to conduct future studies with a wider sample with similar socio-demographic characteristics. Furthermore, we used parents’ reports that present only the parents’ perspective. Further studies should gather information from the children/adolescents as well in order to understand their own experiences and challenges in real life. Finally, we did not analyze the possible confounding effect of psychiatric medications administered to our participants. Future studies should refer to the possible impacts of medications on the results.

## Figures and Tables

**Table 1:** Prevalence of children’s diagnosis (study group).

Psychiatric Diagnosis	Number of Participants (*N* = 25)
Anxiety	6
Anxiety and Attention Deficit Hyperactivity Disorder (ADHD)	10
Anxiety and Oppositional Defiant Disorder (ODD)	1
Anxiety and depression	1
ODD and ADHD	3
Adjustment Disorder and ADHD	2
Depression	1
Dysthymia and depression	1

**Table 2:** Comparing CBCL scores between groups.

	Study Group (*N* = 25) Mean + SE	Control Group (*N* = 24) Mean + SE	F _(1,45)_	η_p_^2^
Anxious/Depressed Withdrawn/Depressed Somatic Complaints	11.31 ± 1.02	1.13 ± 1.08	35.88 ***	0.44
	5.40 ± 0.74	1.69 ± 0.78	9.01 **	0.16
	4.88 ± 0.71	0.90 ± 0.75	11.43 **	0.2
Social Problems	9.17 ± 0.90	0.24 ± 0.95	37.11 ***	0.45
Thought Problems	5.76 ± 0.84	1.17 ± 0.89	10.7 **	0.19
Attention Problems Rule-Breaking Aggressive Behavior	9.87 ± 0.93	1.30 ± 0.98	30.75 ***	0.4
	5.10 ± 0.67	1.10 ± 0.71	12.79 ***	0.22
	13.38 ± 1.53	2.22 ± 1.62	19.26 ***	0.3
Other conditions	6.97 ± 0.94	2.64 ± 1.00	7.57 **	0.14
Internalization	68.23 ± 2.38	47.30 ± 2.51	28.06 ***	0.38
Externalization	62.33 ± 2.50	42.90 ± 2.63	22.00 ***	0.32
Total Problems	67.38 ± 2.81	41.53 ± 2.97	30.63 ***	0.4

Note: SE = standard error; η_p_^2^ (partial η^2^) = effect size; ** *p* ≤ 0.01; *** *p* ≤ 0.001.

**Table 3:** Comparing BRIEF scores between groups.

	Study Group (*N* = 25) Mean + SE	Control Group (*N* = 24) Mean + SE	F _(1,49)_	η_p_^2^
Inhibition	65.78 ± 2.63	43.47 ± 2.70	26.81 ***	0.36
Shifting	72.93 ± 3.0	50.40 ± 3.08	20.98 ***	0.31
Emotional Control	65.99 ± 2.39	45.59 ± 2.45	27.20 ***	0.37
Initiation	64.12 ± 2.51	43.90 ± 2.58	24.17 ***	0.34
Working Memory	65.06 ± 2.28	45.72 ± 2.34	26.84 ***	0.36
Planning	63.38 ± 2.39	43.47 ± 2.46	25.71 ***	0.35
Organizing Materials	53.22 ± 2.51	46.34 ± 2.58	2.78	0.05
Monitoring	62.87 ± 2.27	41.34 ± 2.37	33.35 ***	0.42
BRI	70.38 ± 2.68	45.64 ± 2.75	31.81 ***	0.4
MI	64.08 ± 2.47	43.32 ± 2.54	26.25 ***	0.36
GEC	67.43 ± 2.53	43.63 ± 2.60	32.85 ***	0.41

Note: SE = standard error; η_p_^2^ (partial η^2^) = effect size; BRI = Behavioral Regulation Index; MI = Metacognition Index; GEC = global executive composite; *** *p* < 0.001.

**Table 4:** Comparing Peds-QoL scores between groups.

	Study Group (*N* = 25) Mean + SE	Control Group (*N* = 24) Mean + SE	F _(1,49)_	η_p_^2^
Physical QOL	68.19 ± 5.13	82.29 ± 5.27	2.82	0.05
Emotional QOL	46.59 ± 4.45	85.41 ± 4.57	28.39 ***	0.38
Social QOL	55.31 ± 5.22	99.93 ± 5.36	27.24 ***	0.37
School QOL	51.32 ± 5.19	82.78 ± 5.33	13.68 ***	0.23
Psychosocial QOL	51.08 ± 3.73	89.37 ± 3.83	39.28 ***	0.46
Total QOL	55.35 ± 3.80	87.60 ± 3.90	26.89 ***	0.37

Note: SE = standard error; η_p_^2^ (partial η^2^) = effect size; *** *p* ≤ 0.001.

**Table 5:** The correlations between self-regulation according to the CBCL, EFs according to the BRIEF and QOL according to the Peds-QL in the study group.

		Anxious/ Depressed	Withdrawn/Depressed	Somatic Complaints	Social Problems	Thought Problems	Attention Problems	Rule Breaking	Aggressive Behavior	Other conditions	Internalizing	Externalizing	Total Problems
BRIEF	Inhibition	0.33	0.14	0.13	0.73 ***	0.18	0.65 ***	0.47 *	0.67 ***	0.31	0.28	0.66 ***	0.59 **
	Shifting	0.59 **	0.44 *	0.34	0.52 **	0.33	0.46 *	0.25	0.40 *	0.12	0.59 **	0.43 *	0.59 **
	Emotional control	0.66 ***	0.46 *	0.44 *	0.61 ***	0.32	0.58 **	0.60 **	0.73 ***	0.27	0.65 ***	0.71 ***	0.74 ***
	Initiation	0.56 *	0.58 **	0.33	0.63 ***	0.25	0.59 **	0.36	0.47 *	0.09	0.64 ***	0.53 **	0.65 ***
	Working memory	0.28	0.37	0.39	0.58 **	0.1	0.61 ***	0.19	0.44 *	0.19	0.53 **	0.44 *	0.60 **
	Planning	0.48 *	0.54 **	0.35	0.61 ***	0.27	0.70 ***	0.32	0.48 *	0.13	0.64 ***	0.54 **	0.67 ***
	Organizing materials	0.37	0.62 ***	0.43 *	0.45 *	0.16	0.50 *	0.22	0.39	0.29	0.58 **	0.61	0.53 **
	Monitoring	0.39	0.42 *	0.26	0.71 ***	0.24	0.72 ***	0.38	0.56 **	0.27	0.47 *	0.55 **	0.62 ***
	BRI	0.60 **	0.39	0.35	0.71 ***	0.31	0.65 ***	0.52 **	0.70 ***	0.28	0.57 **	0.69 ***	0.74 ***
	MI	0.49 *	0.59 **	0.40 *	0.68 ***	0.24	0.72 ***	0.36	0.54 **	0.21	0.67 ***	0.57 **	0.70 ***
	GEC	0.60 **	0.56 **	0.42 *	0.76 ***	0.31	0.74 ***	0.46 *	0.66 ***	0.26	0.70 ***	0.68 ***	0.79 ***
Peds-QL	Physical QOL	−0.58 **	−0.69 ***	−0.4	−0.39	−0.29	−0.27	−0.26	−0.35	−0.27	−0.63 ***	−0.34	−0.47 *
	Emotional QOL	−0.60 **	−0.64 ***	−0.59 **	−0.46 *	−0.46 *	−0.47 *	−0.33	−0.50 *	−0.22	−0.76 ***	−0.57 **	−0.69 ***
	Social QOL	−0.42 *	−0.50 *	−0.29	−0.67 ***	−0.28	−0.40 *	−0.45 *	−0.56 **	−0.01	−0.44 *	−0.54 **	−0.58 **

Note: * *p* ≤ 0.05; ** *p* ≤ 0.01; *** *p* ≤ 0.001.

**Table 6:** Predicting QOL domains by EF (study group).

	Model 1				Model 2			
Variables	B	SE B	β	CI (95%)	B	SE B	β	CI (95%)
Physical QOL								
Internalization	−1.69	0.43	−0.63	−2.59–−0.79				
R^2^		0.399						
F_1,23_ for change in R^2^		15.24 ***						
Emotional QOL								
Internalization	−1.47	2.61	−0.76	−2.01–−0.93	−1.08	0.29	−0.56	−1.69–−0.48
BRI					−0.509	0.22	−0.34	−0.97–−0.04
R^2^		0.58				0.66		
F_1,23_ for change in R^2^		31.8 ***				5.18 *		
Social QOL								
MI	−1.3	0.46	−0.508	−2.26–−0.35				
R^2^		0.258						
F_1,23_ for change in R^2^		7.99 **						
School QOL								
MI	−1.65	0.33	−0.72	−2.34–−0.96				
R^2^		0.519						
F_1,23_ for change in R^2^		24.76 ***						
Psychosocial QOL								
MI	−1.30	0.201	−0.80	−1.72–−0.88	−0.79	0.22	−0.48	−1.25–−0.32
Internalization					−0.87	0.25	−0.47	−1.41–−0.34
R^2^		0.646				0.768		
F_1,23_ for change in R^2^		41.91 ***				11.54 **		
Total QOL								
Internalization	−1.53	0.23	−0.80	−2.02–−1.05	−1.16	0.25	−0.61	−1.70–−0.63
BRI					−0.49	0.19	−0.34	−0.90–−0.08
R^2^		0.65				0.72		
F_1,23_ for change in R^2^		43.05 ***				6.30 *		

Note: * *p* ≤ 0.05; ** *p* ≤ 0.01; *** *p* ≤ 0.001.

## Data Availability

Data are not publicly available due to privacy and ethical restrictions.
